# Excision of the Coecum

**Published:** 1885-07

**Authors:** 


### Excision of the Cœcum.

Mr. Walter Whitehead recently excised, at the Manchester Royal Infirmary, the cœcum and the ascending colon of a man suffering from a carcinomatous growth encircling a large extent of the bowel. After the excision, the ileum was attached to the skin below, and the commencement of the transverse colon to the skin above, in the primary incision made through the abdominal wall just outside the rectus. The superior-mesenteric vein was wounded during the removal of an infiltrated mesenteric gland, and had to be ligatured. The operation, which was conducted on Listerian principles, occupied nearly two hours, and was found to be more tedious than difficult.

We learn that four days after the operation the patient was free from a single untoward symptom, and promises to make a complete recovery, the temperature never once having exceeded normal limits.—*British Medical Journal.*

